# The Relationship between Handgrip Strength, Timed Up-and-Go, and Mild Cognitive Impairment in Older People during COVID-19 Pandemic Restrictions

**DOI:** 10.3390/bs13050410

**Published:** 2023-05-14

**Authors:** Jiranan Griffiths, Mathuramat Seesen, Wachiranun Sirikul, Penprapa Siviroj

**Affiliations:** 1 Department of Occupational Therapy, Faculty of Associated Medical Sciences, Chiang Mai University, Chiang Mai 50200, Thailand; jiranan.gr@cmu.ac.th; 2 Department of Community Medicine, Faculty of Medicine, Chiang Mai University, Chiang Mai 50200, Thailand; mathuramat.s@cmu.ac.th (M.S.); wachiranun.sir@cmu.ac.th (W.S.); 3 Center of Data Analytics and Knowledge Synthesis for Health Care, Chiang Mai University, Chiang Mai 50200, Thailand

**Keywords:** mild cognitive impairment, Montreal Cognitive Assessment Basic, handgrip strength, Timed Up-and-Go, older adults, COVID-19 restrictions

## Abstract

The COVID-19 lockdown restrictions affected physical performance and cognitive function in older people as they were confined to their homes. There is an association between physical and cognitive functions. Mild Cognitive Impairment (MCI) is a condition that risks progressing to dementia. This study aimed to identify the relationship between handgrip strength (HGS), Timed Up-and-Go (TUG), and MCI in older people during the COVID-19 pandemic restrictions. The cross-sectional study recruited 464 eligible participants for an interview and anthropometric measurement. The Montreal Cognitive Assessment-Basic (MoCA-B), HGS, and TUG were measured in addition to demographic and health characteristics. A total of 398 participants (85.8%) were found to have MCI when screened with the MoCA-B. Their mean age was 71.09 ± 5.81 years. Forward multiple regression analysis demonstrated that HGS (β = 0.032, *p* < 0.001), education level (β = 2.801, *p* < 0.001), TUG (β = −0.022, *p* = 0.013), Thai Geriatric Depression Score, TGDS (β = −0.248, *p* = 0.011), and age (β = −1.677, *p* = 0.019) were associated with MCI. A decrease in HGS and an increased TUG might allow for the early detection of MCI and promote physical training in order to reduce the risk of MCI. Further studies can investigate multidomain indicators for MCI, for example, fine motor skills and pinch strength as components of the motor abilities.

## 1. Introduction

Mild cognitive impairment (MCI) is a condition that affects cognitive functions such as language, visuospatial, memory, and frontal executive functions [1]. The prevalence of MCI has varied depending on the methods of screening or the diagnosis process, areas of study, and the different participants’ characteristics. One study showed that the prevalence of MCI worldwide ranged from 5–36.7%, which included the USA, Europe (e.g., UK, France, Italy, and Spain), Asia (e.g., Hong Kong and Singapore), and Australia [2]. In Thailand, a high prevalence of MCI was found in older people in a rural community of 71.4% [3]. People who have MCI are often found to have a slight deficit in the performance of the instrumental activities of daily living (IADL), but the basic activities of daily living (BADL) remain independent. It is possible for MCI to progress to dementia [4]. 

Identifying the indicators of MCI may help in the early detection of people who are at risk so that support can be given at an early stage to reduce the chance that the MCI will progress to dementia [5]. A previous study reported that the Montreal Cognitive Assessment meets the criteria for screening tests for the detection of MCI in people over 60 years of age and is better than the Mini-Mental State Examination (MMSE) [6]. The Montreal Cognitive Assessment-Basic (MoCA-B) is one of the screening tools that has been developed for screening for MCI in older people with low education and literacy. It has been developed based on the original MoCA, which was validated in people who had an education of approximately 13 years. Low education and literacy may influence cognitive performance; therefore, the MoCA-B has been validated in people with an education of less than 5 years [7,8].

The evidence shows that there is an association between physical and cognitive function. Low physical activity increases the risk of MCI and dementia [9]. Physical frailty is one of the geriatric syndromes that can develop into dependency and/or mortality and is a predictor of disability [10]. Older people who are physically frail and also have cognitive impairment are considered to be cognitively frail [11]. Handgrip strength (HGS) and walking speed are used as part of the criteria to measure physical frailty in older people using a modified Fried frailty phenotype measure [12]. Studies have reported the association between HGS and the risk of cognition decline [13,14]. Weaker handgrip is found to be associated with cognitive decline over a 7 year period [15]. This suggests that measuring grip strength may be a simple and inexpensive method to identify people who are at risk of cognitive decline. Similarly, people with MCI exhibit more gait and balance impairment [16]. Timed Up-and-Go (TUG) is one of the tests that can be used to assess mobility impairment. TUG can be conducted quickly and it is widely used [17,18,19]. It may be useful as a marker for the early detection of cognitive impairment.

Likewise, there are several common risk factors for cognitive and physical impairment in older people that may increase their risk of developing these diseases. These factors can be evaluated using screening tools, including poor nutrition using the Mini Nutritional Assessment-Short Form (MNA-SF) tool [20,21], difficulties with the daily activities using the ADL assessment tool [22,23,24], depression using the Geriatric Depression Scale (GDS) [25,26,27], and poor sleep quality using the Pittsburgh Sleep Quality Index (PSQI) [28,29]. Preventing or delaying cognitive decline in older persons may be practical with the early detection of these risk factors.

During the COVID-19 pandemic, the lockdown or activity restrictions were in place for more than two years to protect the vulnerable populations who were at high risk [30,31]. As a result, older people spent a long time at home. This worsened physical performance and induced muscular atrophy of the extremities [32]. There is a lack of evidence on cognitive decline among older Thai people in the community in relation to physical parameters such as handgrip strength and TUG. During the restrictions enforced by the nationwide lockdown following the second COVID-19 pandemic in Thailand, we conducted this study on older Thai people who lived in the community and were exposed to the restrictions (June to December 2021) [33]. This study aimed to identify a relationship between HGS and TUG and MCI in older adults, aged over 65 years and living in northern Thailand. 

## 2. Methods

### 2.1. Study Design and Participants

**Figure 1:**
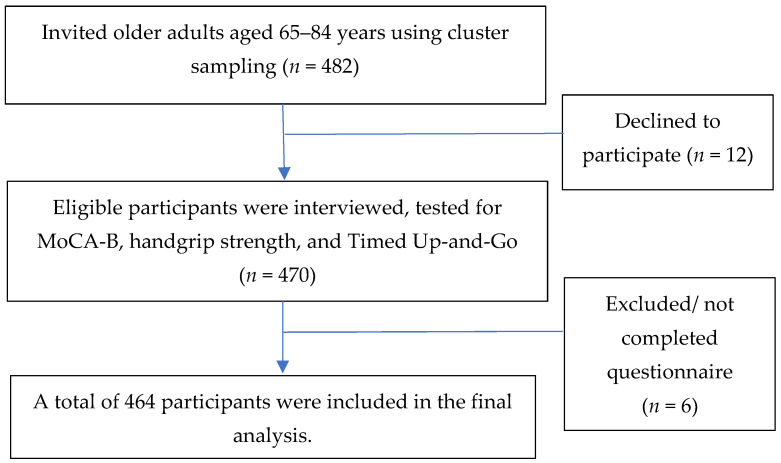
Diagram of the study participant selection.

The methods and results of this study are reported in accordance with the Strengthening the Reporting of Observational Studies in Epidemiology (STROBE) guidelines for cross-sectional studies [34]. The participant recruitment, sampling design, and inclusion and exclusion criteria can be found in our previous study [21]. We briefly provide them again in this study. The cross-sectional study was conducted in Khua Mung Subdistrict, Saraphi District, Chiang Mai Province, Thailand, in July 2021. Sample size was calculated using EpiInfoTM version 7.2 [35] based on the population survey or descriptive study. We used the population size of 934 persons. The expected frequency using the prevalence of cognitive impairment was 10.9% [36]. A confidence interval of 97%, an acceptable margin of error of 4%, and a design effect of 2.0 was set. The total sample size was determined to be 438. We aimed for 10% oversampling, and thus a minimum of 482 people were needed. We used cluster sampling methods in ten villages to recruit the participants. The self-reported history of disease was confirmed with medical records in the health-promoting hospital database. We excluded those who had been diagnosed with dementia, depression, end-stage kidney disease, hepatitis, cirrhosis, autoimmune diseases, cancer, acute trauma, acute illnesses, and those who took steroids. In total, 464 participants were included in the data analysis (Figure 1).

### 2.2. Data Collection and Measurement

Assessments of the participants using MoCA-B were conducted by ten medical students who were trained and supervised to screen for MCI by an occupational therapist who had the Montreal Cognitive Assessment (MoCA) certification (The certification number was THGRIJI69617-02 and it was given by Dr. Nasreddine Ziad). 

A questionnaire was used to collect information on demographic and health characteristics, such as age, sex, marital status, education level, living situation, smoking and alcohol use, as well as the participant’s history of hypertension, type 2 diabetes mellitus, dyslipidemia, coronary artery disease, stroke, gout, asthma, chronic obstructive pulmonary disease (COPD), and chronic kidney disease. The participants were interviewed for BADL [37]. Depression was estimated using the Thai Geriatric Depression Scale (TGDS) [25,38]. The Mini Nutritional Assessment-Short Form (MNA-SF) [39,40], Pittsburgh Sleep Quality Index (PSQI) [28,41], Montreal Cognitive Assessment-Basic (MoCA-B) [7,8], Handgrip Strength (HGS), Timed Up-and-Go (TUG), and Body Mass Index (BMI) were also evaluated. 

We used the Thai version of the Montreal Cognitive Assessment-Basic (MoCA-B), which was able to identify MCI in individuals who had a low level of education and literacy; literacy-dependent tasks were eliminated. The MoCA-B was validated among community-dwelling elderly Thai persons with low education levels and demonstrated excellent discrimination performance for MCI screening (cutoff score of 24, 81% sensitivity and 86% specificity) [8]. The maximum score was 30. For MCI, the cutoff score was ≤24. For individuals who had <4 years of education, one point was added to the overall score and two points were added for individuals who had <4 years of education and were illiterate. Illiteracy was defined as the inability to read or write fluently in daily living. 

Handgrip strength (HGS) was measured using a digital standard dynamometer (T.K.K.5401 GRIP D; Takei Scientific Instruments Co., Ltd., Niigata, Japan). Two measurements were obtained for each hand and the higher value was used [42]. According to the Asian Working Group for Sarcopenia, low grip strength was defined as <28 kg in males and <18 kg in females [43]. We also calculated the %HGS normalized for 28 kg in males and 18 kg in females.

To perform the TUG test, the participants were first asked to stand from a seated position, walk at a comfortable and safe pace to a line on the floor three meters away, turn and walk back to the chair and sit down again [44,45]. TUG in this study adopted the age-specific reference values proposed in a meta-analysis: 9.0 s (65–69 years old), 10.2 s (70–79 years old), and 12.7 s (≥80 years old). Using these cutoff values, subjects were classified as better or worse than the age-specific cutoff value [46]. 

### 2.3. Statistical Analysis

The data were analyzed using the IBM SPSS statistical package version 28.0 for Windows (IBM Corp., Armonk, NY, USA). The data were expressed as mean ±SD for normally distributed continuous values (Shapiro–Wilk test), and median [IQR] for non-normally distributed continuous variables. Demographic and health characteristics were reported as frequencies and with percentages. The comparison of mean value of HGS and TUG between non-MCI and MCI was performed using independent sample *t*-test and Mann–Whitney U test. The association between HGS, TUG, and cognitive function as MoCA-B scores and non-MCI and MCI were analyzed with Pearson correlation and Chi-square tests. Simple linear regression was used to test the association between HGS, TUG, and MoCA-B with pre-defined, potentially associated factors as confounders, e.g., demographic and health characteristics, MNA-SF, ADL, TGDS, and PSQI. Forward multiple linear regression was performed to analyze the association of significant factors in simple linear regression. All statistical tests were two-sided, and a *p*-value of 0.05 or lower was regarded as statistically significant.

## 3. Results

### 3.1. Participants’ Characteristics 

A total of 464 older adults were recorded. Table 1 shows the participants’ characteristics. The mean age was 70.76 ± 5.59 years. A total of 58.4% of the participants were female. They had a low level of education (1–3 years (36.6%) or 4–6 years (49.8%), and 93.1% of them did not smoke. Only 55 participants (11.8%) had three or more chronic diseases. There were no significant differences in the baseline characteristics between the non-MCI and MCI groups except age, marital status, and education level. The screening for the score of parameters, such as MNA-SF, BMI, ADL, TGDS, and PSQI, showed that there were no significant differences between the two groups.

### 3.2. MoCA-B Scores and Difference between the Non-MCI and MCI Groups

A total of 398 participants (85.8%) were identified as having MCI, with a MoCA-B score of ≤24 (Table 2). The median and interquartile range for all participants’ MoCA-B scores was 20.0 [7.0]. There was a significant difference in the MoCA-B median scores between the non-MCI (median = 26.0, IQR = 2.25) and MCI groups (median = 19.0, IQR = 6.0) (Table 2). Figure S1 shows that the highest failure of cognitive function impairments in each domain among all participants were executive function (94.5%), alternating attention (45.2%), delayed recall (44.5%), and attention (40.5%), respectively. The Figure S2 histogram shows that for all participants, the MoCA-B scores ranged between 4 and 30 points, and the mean ±SD was 19.35 (±4.86).

### 3.3. Handgrip Strength and Timed Up-and-Go

The average HGS in males was 24.70 (±7.49) kg and in females was 16.46 kg (±4.67). Between the non-MCI and MCI groups, the mean percent HGS was significantly different (*p* < 0.001) (Table 2). There were also significant differences in the percent HGS below 100% (poor HGS) between the MCI (86.6%) and non-MCI group (71.2%) (*p* < 0.001) (Figure S3). The average TUG (s) for all participants aged 65–69 years, 70–79 years, and ≥80 years was 11.38 (±2.70), 12.83 (±3.65), and 14.20 (±3.61), respectively. There was a significantly different mean percent TUG between the MCI and non-MCI groups (*p* < 0.001) (Table 2). The MCI group’s percent TUG > 100% (bad TUG) was significantly lower than that of the non-MCI group (*p* = 0.008) (Figure S3).

### 3.4. Relationship between Handgrip Strength, Timed Up-and-Go, and Mild Cognitive Impairment

**Figure 2:**
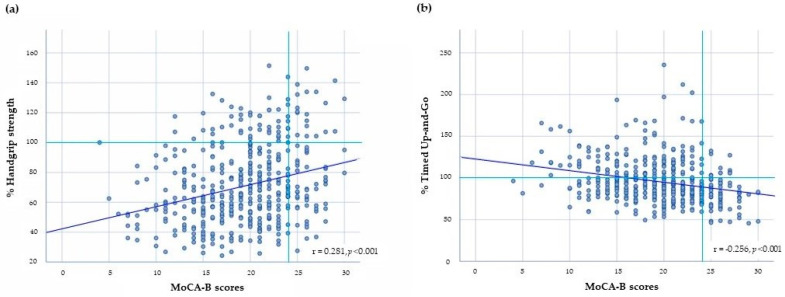
The relationship between (**a**) % Handgrip Strength (HGS) and Montreal Cognitive Assessment-Basic (MoCA-B) scores, and (**b**) % Timed Up-and-Go (TUG) and MoCA-B scores; Significant *p*-values were analyzed using Pearson correlation coefficient.

The MoCA-B scores were positively associated with the percent HGS (r = 0.281, *p* < 0.001) (Figure 2a), while they were negatively associated with the percent TUG (r = −0.256, *p* < 0.001) (Figure 2b). In Figure S3, 86.6% of the participants in the MCI group had a percent HGS < 100% (decreased HGS) and 35.9% had a percent TUG ≥ 100% (increased TUG). In the non-MCI group, 71.2% had a percent HGS < 100% (decreased HGS), and 16.7% had a percent TUG ≥ 100% (increased TUG).

### 3.5. The Factors Associated with MoCA-B Scores

Table 3 presents the factors associated with the MoCA-B scores when using simple linear regression analysis. The following results were all found to be associated with the MoCA-B scores: age (β = −2.987, *p* < 0.001), marital status (β = −1.004, *p* = 0.030), education level (β = 4.139, *p* < 0.001), MNA-SF score (β = 0.137, *p* = 0.003), TGDS score (β = −0.1404, *p* < 0.001), percent HGS (β = 0.053, *p* < 0.001), and percent TUG (β = −0.047, *p* < 0.001).

A forward multiple linear regression was performed on the full exploratory model, to identify the factors associated with the MoCA-B scores using the factors that were found to be significant in the simple linear regression. The data were tested for the assumption of collinearity and indicated that multicollinearity was not a concern (Table S1). Age, marital status, education level, MNA-SF score, TGDS score, percent HGS, and percent TUG were entered. The results showed that the MoCA-B score and all factors except marital status and MNA-SF were significantly associated. The significance of these factors in descending order was: percent HGS (β = 0.032, *p* < 0.001), education level (β = 2.801, *p* < 0.001), percent TUG (β = −0.022, *p* = 0.013), TGDS score (β = −0.248, *p* = 0.011), and age (β = −1.677, *p* = 0.019). Approximately 15.5% of the variance in the MoCA-B scores was explained by these factors (adjusted R^2^ = 0.155, *p* < 0.001) (Table 4).

## 4. Discussion

Mild cognitive impairment (MCI) is a syndrome that risks progressing to dementia in older people [47]. Older people tend to become more inactive as they age, resulting in a deterioration in their physical condition and cognitive function [48,49]. The prevalence of MCI in our study was high at 85.8% and low education was associated with MCI. This was consistent with a previous study, which used the MoCA-B as a screening tool in a rural community in Thailand and found a high prevalence of MCI of 71.4%. The authors found that a low education and diabetes mellitus were significant risk factors [3]. Our study found that there was no difference between males and females in the MCI and non-MCI groups. We excluded older people with depression as depression is commonly found in MCI. Another study reported that the prevalence of depression in MCI ranged between 16.9–55% [50]. 

Identifying the physical indicators of MCI helps to enable screening and allows preventive intervention programs to support the independence of people at risk of dementia. This study determined that HGS and TUG, which are widely used in research and clinical settings to assess the strength of the handgrip and the balance from sitting to standing as indicators of cognitive function. Using the MoCA-B to screen for MCI in this study showed that alternating attention, delayed recall, and executive function were the most common problems. Our study found that the MoCA-B score was associated with HGS and TUG. In addition, the HGS and TUG scores in the MCI group were significantly poorer than in the non-MCI group. HGS in the MCI group was significantly lower than in the non-MCI group and the TUG in the people with MCI was significantly longer than in the people with non-MCI. These studies demonstrated that there was an association between HGS, functional mobility, and executive function [48,51].

Our study showed that HGS and TUG were physical indicators of cognitive impairment. We found that approximately 15.5% of the variance related to cognitive impairment was explained by the handgrip strength and TUG measures. This was consistent with previous studies showing that low handgrip strength was associated with a risk of cognitive decline [14,15,52,53,54]. HGS could be one of the indicators for cognitive impairment. HGS was found to be associated with cognition especially in executive function tasks [55,56,57]. Our study found that the association between cognitive function and TUG was consistent with previous studies [58,59,60]. TUG measures have been associated with lower levels of cognition. TUG is longer in older people with mild–moderate cognitive impairment or those with Parkinson’s disease. The TUG test is a particularly suitable tool for detecting subtle movement deficits [61].

According to previous studies, the correlation between poor physical condition and cognitive decline might be explained by reduced gray and white matter volumes in multiple brain regions. Muscle strength, volumetric brain measures, and the reduction in handgrip strength have been linked to age markers in the brain [62]. A study explored whether muscle strength, mainly handgrip strength and gait speed, were linked to brain volumetrics and white matter hyperintensity accumulation. The authors found some association between HGS and white matter volume and reported evidence that gait speed was positively related to the whole brain volume [63]. The networks in the brain that control walking involve areas for attention, executive function, and visuospatial functions, as well as motor task control. The cerebellum, basal ganglia, hippocampus, and parietal and frontal cortices have all been linked to executive processes and gait in previous studies [64,65]. Another study found that gait and walking were related to cognitive function, and a decline in gait speed occurred earlier in men than in women [66].

There is little literature on the association between HGS and TUG on cognition decline using the MoCA-B during the COVID-19 restrictions in Thailand. The COVID-19 pandemic has had a significant impact on older adults, particularly in terms of physical activities. Older adults were advised or required to socially distance. This limited their opportunities for social engagement, physical activity, and access to healthcare [67,68]. Older adults with poorer HGS and TUG showed a higher risk of developing MCI. There may be a potential to use muscle strength and mobility tests to identify MCI in older persons because these indicators typically appear considerably earlier than a decline in cognitive function. It is hoped that these simple tests can indicate a difference between those with and without MCI. In addition, future studies with a broader range of cognitive functions and other physical functions are needed to better describe the associations between cognitive and physical function. Other indicators should be investigated to evaluate the common mechanisms for the decline in both cognitive and physical functions. HGS and TUG can be a used to screen as risk factors of MCI and the information can be used to promote physical activities in order to reduce the risk of MCI and dementia. Muscle strength and balance are associated with cognitive functions. This association suggests that there may be potential benefits to physical training and physical activities, such as hand strength training, balance training, and that functional mobility may delay mild cognitive impairment.

This study was strengthened using cluster sampling of the participants. Moreover, we excluded participants who were diagnosed with dementia and depression by doctors, which could cause an incorrect diagnosis of cognitive impairment. However, there were limitations. Firstly, a causal relationship could be established as this was a cross-sectional study. Secondly, the results might not be generalizable to other settings due to the limitations of using data from one sub-district. Thirdly, we did not measure physical activity due to the COVID-19 pandemic restrictions that limited the activities of the participants.

## 5. Conclusions 

Our study shows evidence that HGS and TUG are associated with MCI measured using the MoCA-B in community-dwelling older adults, aged 65–84 years. Given that there is a bidirectional relationship between physical and cognitive functioning, we suggest that these two parameters may be used to screen in order to provide programs that may help in delaying MCI and dementia. Primary care units should establish physical training to delay MCI as early as possible. In the future, it will be useful to screen multidomain parameters to identify the stronger indicators of MCI. Further studies should also identify the fine motor skills and pinch strength as components of the motor abilities to determine if they are associated with a decline in cognitive function.

## Figures and Tables

**Table 1:** Participants’ characteristics by subjective cognitive decline status.

Variables	*n* (%)			*p*-Value
	Total (*n* = 464)	Non-MCI (*n* = 66)	MCI (*n* = 398)	
Age (years), mean ±SD	70.67 ± 5.59	68.18 ± 3.10	71.09 ± 5.81	
65–69	245 (53.0)	52 (78.8)	193 (48.7)	<0.001 ^a^
70–79	170 (36.8)	14 (12.2)	156 (39.4)	
≥80	47 (10.2)	0 (0.0)	47 (11.9)	
Sex				
Male	193 (41.6)	31 (47.0)	162 (40.7)	0.348 ^a^
Female	271 (58.4)	35 (53.0)	236 (59.3)	
Marital status				
Married	283 (61.0)	49 (74.2)	234 (58.8)	0.020 ^a^
Single/divorced/widowed	181 (39.0)	17 (25.8)	164 (41.2)	
Education level				
No education	13 (2.8)	2 (3.0)	11 (2.8)	<0.001 ^a^
Primary school (1–3 years)	170 (36.6)	18 (27.3)	152 (38.2)	
Primary school (4–6 years)	231 (49.8)	22 (33.3)	209 (45.0)	
Secondary school (12 years)	34 (7.3)	15 (22.7)	19 (4.8)	
Bachelor’s degree (>12 years)	16 (3.4)	9 (13.6)	7 (1.8)	
Living status, alone	58 (12.5)	7 (10.6)	51 (12.8)	0.693 ^a^
Current smoking	32 (6.9)	4 (6.1)	28 (7.1)	0.713 ^a^
Current drinking	69 (14.9)	15 (22.7)	54 (13.6)	0.062 ^a^
Numbers of chronic diseases				
0	134 (28.9)	19 (28.8)	115 (85.8)	0.944 ^a^
1–2	275 (59.3)	40 (60.6)	235 (85.5)	
≥3	55 (11.8)	7 (10.6)	48 (12.1)	
Hypertension	237 (51.1)	31 (47.0)	206 (51.8)	0.508 ^a^
Dyslipidemia	79 (17.0)	12 (18.2)	67 (16.8)	0.860 ^a^
Type 2 Diabetes mellitus	78 (16.8)	13 (19.7)	65 (16.3)	0.594 ^a^
Gout	20 (4.3)	4 (6.1)	16 (4.0)	0.507 ^a^
Coronary heart disease	18 (3.9)	3 (4.5)	15 (3.8)	0.762 ^a^
Asthma	15 (3.2)	2 (3.0)	13 (3.3)	1.000 ^a^
Stroke	14 (3.0)	2 (3.0)	12 (3.0)	1.000 ^a^
Chronic kidney disease	12 (2.6)	1 (1.5)	11 (2.8)	0.708 ^a^
COPD	7 (1.5)	0 (0.0)	7 (1.5)	0.600 ^a^
MNA-SF (points), median [IQR]	10.15 ± 2.04	10.48 ± 1.73	10.18 ± 2.04	
Normal	138 (29.7)	21 (31.8)	117 (29.4)	0.527 ^a^
At risk of malnutrition	271 (58.4)	40 (60.6)	231 (58.0)	
Malnourished	55 (11.9)	5 (7.6)	50 (12.6)	
BMI, median [IQR]	22.75 ± 3.92	23.63 ± 4.46	22.61 ± 3.80	
Underweight (<18.5 kg/m^2^)	54 (12.2)	5 (7.6)	49 (13.0)	0.402 ^a^
Normal weight (18.5–22.9 kg/m^2^)	191 (43.0)	27 (40.9)	164 (43.4)	
Overweight (23.0–24.9 kg/m^2^)	89 (20.0)	13 (19.7)	76 (20.1)	
Obese (>25.0 kg/m^2^)	110 (24.8)	21 (31.8)	89 (23.5)	
ADL (points), median [IQR]	20.0 [0.0]	20.0 [0.0]	20.0 [0.0]	0.147 ^b^
TGDS (points), median [IQR]	2.0 [2.0]	1.0 [3.0]	2.0 [2.0]	
Depression	61 (13.1)	4 (6.6)	57 (93.4)	1.000 ^a^
PSQI (points), median [IQR]	5.0 [3.0]	8.0 [2.5]	8.0 [3.0]	
Sleep quality, bad	231 (49.8)	34 (14.7)	197 (85.3)	0.865 ^a^

Non-MCI, normal cognitive function; MCI, Mild Cognitive Impairment; COPD, Chronic Obstructive Pulmonary Disease; MNA-SF, Mini Nutritional Assessment-Short Form; BMI, Body Mass Index; ADL, Activities of Daily Living; TGDS, Thai Geriatric Depression Scale; PSQI, Pittsburgh Sleep Quality Index; SD, Standard Deviation; IQR, Interquartile Range; Significant *p*-values were analyzed using ^a^ Chi-square test, and ^b^ Mann–Whitney U test.

**Table 2:** MoCA-B scores, handgrip strength and Timed Up-and-Go between non-MCI and MCI groups.

Parameters	Mean ±SD, Median [IQR]						*p*-Value
	*n*	Total (*n* = 464)	*n*	Non-MCI (*n* = 66, 14.2%)	*n*	MCI (*n* = 398, 85.8%)	
MoCA-B scores	464	19.35 ± 4.86 20.00 [7.00]		26.41 ± 1.40 26.00 [2.25]		18.18 ± 4.19 19.0 [6.00]	<0.001 ^a^
HGS (kg)	461	19.91 ± 7.26, 18.60 [9.70]	66	23.42 ± 8.32, 22.50 [12.52]	395	19.33 ± 6.90, 17.90 [9.30]	<0.001 ^b^
Male	193	24.70 ± 7.49, 25.40 [11.00]	31	29.80 ± 7.03, 30.00 [9.00]	162	23.73 ± 7.20, 24.05 [11.20]	<0.001 ^b^
Female	268	16.46 ± 4.67, 16.15 [6.60]	35	17.78 ± 4.36, 17.60 [5.90]	233	16.26 ± 4.69, 16.00 [6.45]	0.034 ^a^
Percent HGS	459	71.12 ± 25.92, 66.43 [34.64]	66	83.65 ± 29.73, 80.34 [44.73]	395	69.02 ± 24.66, 63.93 [33.21]	<0.001 ^b^
Male	193	88.23 ± 26.76, 90.71 [32.29]	31	106.42 ± 25.10, 107.22 [32.14]	162	84.75 ± 25.71, 85.89 [40.0]	<0.001 ^b^
Female	268	58.79 ± 16.69, 57.68 [23.57]	35	63.49 ± 15.59, 62.85 [21.07]	233	58.09 ± 16.77, 57.14 [23.04]	0.034 ^a^
TUG (s)	459	12.20 ± 3.33, 11.58 [3.73]	66	10.36 ± 2.53, 10.26 [3.40]	393	12.50 ± 3.35, 11.79 [4.03]	<0.001 ^b^
65–69 years old	244	11.38 ± 2.70, 11.03 [3.06]	52	10.35 2.50, 10.26 [3.31]	192	11.65 ± 2.69, 11.21 [2.95]	0.003 ^a^
70–79 years old	169	12.83 ± 3.65, 12.07 [3.96]	14	10.38 ± 2.74, 10.38 [5.21]	155	13.06 ± 3.65, 12.26 [4.12]	0.012 ^a^
≥80 years old	44	14.20 ± 3.61, 13.86 [4.24]	0	-	44	14.20 ± 3.61, 13.86 [4.24]	-
Percent TUG	459	96.03 ± 26.21, 91.18 [29.37]	66	81.54 ± 19.95, 80.83 [26.77]	393	98.46 ± 26.37, 92.83 [31.69]	<0.001 ^b^
65–69 years old	244	89.58 ± 21.27, 86.85 [24.11]	52	81.49 ± 19.71, 80.83 [26.04]	192	91.77 ± 21.19, 88.31 [23.27]	0.003 ^a^
70–79 years old	169	101.06 ± 28.74, 95.04 [31.14]	14	81.72 ± 21.60, 81.77 [40.98]	155	102.81 ± 28.72, 96.53 [32.44]	0.012 ^a^
≥80 years old	44	111.83 ± 28.44, 109.13 [33.37]	0	-	44	111.83 ± 28.44, 109.13 [33.37]	-

SD, Standard Deviation; IQR, Interquartile Range; % Handgrip strength (HGS) were calculated as follows: HGS [kg] × 100/28 (males), HGS [kg] × 100/18 (females). 100% Timed Up-and-Go (TUG) was determined as 9.0 s (65–69 years old), 10.2 s (70–79 years old), and 12.7 s (≥80 years old). Non-MCI, normal cognitive function as Montreal Cognitive Assessment-Basic (MoCA-B) scores >24; MCI, Mild Cognitive Impairment as MoCA-B scores ≤24. Differences in HGS and TUG were analyzed using ^a^ Mann–Whitney U test and ^b^ independent sample *t*-test.

**Table 3:** Factors associated with mild cognitive impairment using MoCA-B score in older people.

Variables	β	95% CI	*p*-Value
Age ≥80 years	−2.987	−4.424 to −1.550	<0.001 **
Sex, Female	−0.630	−1.529 to 0.268	0.169
Marital status, single/divorced/widowed	−1.004	−1.909 to −0.99	0.030 *
Education level, above secondary school	4.139	2.759 to 5.519	<0.001 **
Living status, alone	−0.495	−1.836 to 0.846	0.468
Number of chronic diseases	0.066	−0.343 to 0.474	0.752
Mini Nutritional Assessment-Short Form, MNA-SF (score)	0.137	0.110 to 0.541	0.003 *
Activities of Daily Living, ADL (score)	0.351	−0.155 to 0.856	0.174
Thai Geriatric Depression Scale, TGDS (score)	−0.404	−0.607 to −0.202	<0.001 **
Global Pittsburgh Sleep Quality Index, PSQI (score)	0.001	−0.180 to 0.183	0.990
Percent Handgrip Strength, %HGS	0.053	0.036 to 0.069	< 0.001 **
Percent Timed Up-and-Go, %TUG	−0.047	−0.063 to −0.031	<0.001 **

β, Beta coefficient; CI, confidence interval; MoCA-B, Montreal Cognitive Assessment-Basic; Significant *p*-values were analyzed using simple linear regression; * Significant association at *p* < 0.05, ** Significant association at *p* < 0.001.

**Table 4:** The exploratory model of factors associated with mild cognitive impairment using MoCA-B scores in older people.

Variables	β	95% CI	*p*-Value
Percent Handgrip strength, % HGS	0.032	0.015 to 0.048	<0.001 **
Education level, above secondary school	2.801	1.464 to 4.138	<0.001 **
Percent Timed Up-and-Go, %TUG	−0.022	−0.039 to −0.005	0.013 *
Thai Geriatric Depression Scale, TGDS (point)	−0.248	−0.440 to −0.057	0.011 *
Age ≥80 years	−1.677	−3.080 to −0.273	0.019 *
Constant: Beta = 18.642; 95% CI = 15.514 to 21.770; *p* < 0.001 **			
R squared = 0.164; Adjusted R squared = 0.155; F = 17.627; *p* < 0.001 **			

β, Beta coefficient; CI, confidence interval; MoCA-B, Montreal Cognitive Assessment-Basic; MNA-SF, Mini Nutritional Assessment-Short Form; Significant *p*-values were analyzed by stepwise multiple linear regression after adjustment for marital status and MNA-SF. * Significant association at *p* < 0.05, ** Significant association at *p* < 0.001.

## Data Availability

The data presented in this study are available from the corresponding author on reasonable request.

## Supplementary Materials

The following supporting information can be downloaded at: https://www.mdpi.com/article/10.3390/bs13050410/s1, Figure S1: The percentage of cognitive impairment in each domain of the Montreal Cognitive Assessment-Basic (MoCA-B) among all participants (n = 464); Figure S2: Histogram illustrates (a) the Handgrip strength (HGS), 100% muscle strength was determined as 28 kg for males and 18 kg for females; (b) the Timed Up-and-Go (TUG), 100% TUG was determined as 9.0 s (65–69 years), 10.2 s (70–79 years), and 12.7 s (≥80 years); (c) MoCA-B, Montreal Cognitive Assessment-Basic; MoCA-B scores, normal cognitive function MoCA-B scores >24; MCI, mild cognitive impairment MoCA-B scores ≤24. Figure S3: The differences in the prevalence of percent HGS and percent TUG in non-MCI and MCI groups; Table S1: Correlations among scores of MoCA-B, MNA-SF, ADL, TGDS, PSQI, HGS, and TUG.
