# Supplementary material for: The Relationship between Handgrip Strength, Timed Up-and-Go, and Mild Cognitive Impairment in Older People during COVID-19 Pandemic Restrictions
Source: Behav Sci (Basel). 2023 May 14;13(5):410. doi: 10.3390/bs13050410 (PMC10215916; doi:10.3390/bs13050410)
Supplement: Supplementary file 1 [file behavsci-13-00410-s001.zip › Table S1 new.pdf]

Table S1. Correlations among scores of MoCA-B, MNA-SF, ADL, TGDS, PSQI, HGS and TUG.

	**1**	**2**	**3**	**4**	**5**	**6**	**7**
1. MoCA-B	–						
2. MNA-SF	0.137**	–					
3. ADL	0.063	0.026	–				
4. TGDS	-0.179**	-0.198**	-0.147**	–			
5. PSQI	0.001	-0.130**	-0.089	0.0260**	–		
6. percent HGS	0.281**	0.208**	0.051	-0.109*	-0.158**	–	
7. percent TUG	-0.256**	-0.077	-0.105*	0.185**	0.064	-0.341**	–

MoCA-B), Montreal Cognitive Assessment-Basic; MNA-SF, Mini Nutritional Assessment-Short Form; ADL, Activities of Daily Living; TGDS, Thai Geriatric Depression Scale; PSQI, Pittsburgh Sleep Quality Index; HGS, Handgrip Strength; TUG, Timed Up-and-Go; * p <0.05; ** p <0.01
