# Supplementary figures and images for: The Relationship between Handgrip Strength, Timed Up-and-Go, and Mild Cognitive Impairment in Older People during COVID-19 Pandemic Restrictions
Source: Behav Sci (Basel). 2023 May 14;13(5):410. doi: 10.3390/bs13050410 (PMC10215916; doi:10.3390/bs13050410)

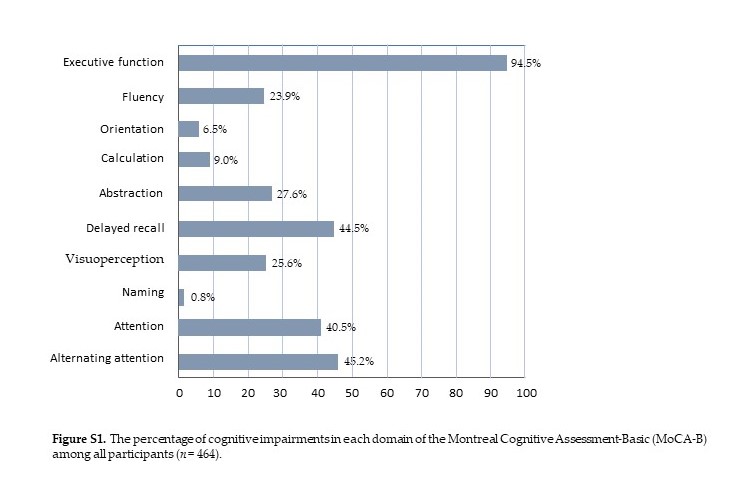

Supplement: Supplementary file 1 [file behavsci-13-00410-s001.zip › Figure S1 new.jpg]

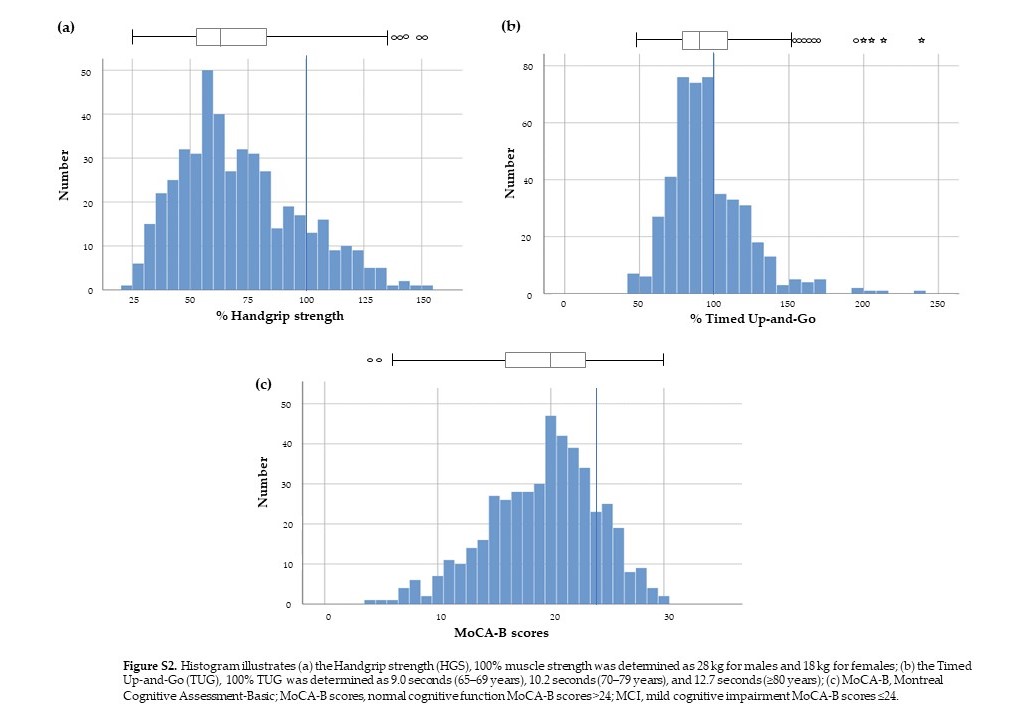

Supplement: Supplementary file 1 [file behavsci-13-00410-s001.zip › Figure S2 new.jpg]

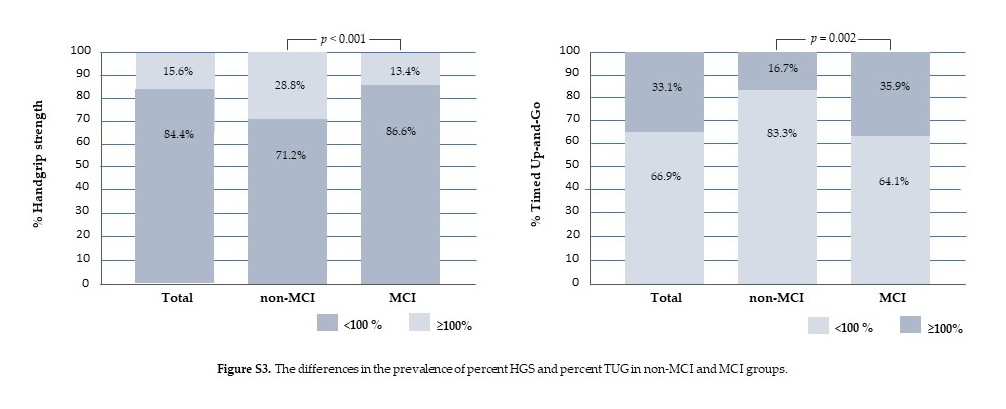

Supplement: Supplementary file 1 [file behavsci-13-00410-s001.zip › Figure S3 new.jpg]
